# Generative Adversarial Networks for Creating Synthetic Nucleic Acid Sequences of Cat Genome

**DOI:** 10.3390/ijms23073701

**Published:** 2022-03-28

**Authors:** Debapriya Hazra, Mi-Ryung Kim, Yung-Cheol Byun

**Affiliations:** 1 Department of Computer Engineering, Jeju National University, Jeju 63243, Korea; debapriyah@jejunu.ac.kr; 2 Veterinary Internal Medicine, Kyungpook National University, Daegu 41566, Korea; karimaker@hanmail.net; 3 Department of Computer Engineering, Institute of Information Science & Technology, Jeju National University, Jeju 63243, Korea

**Keywords:** synthetic genome, generative adversarial networks, nucleic acid sequences, WGAN-GP, cat genome, promoter prediction, promoter classification, motif matching

## Abstract

Nucleic acids are the basic units of deoxyribonucleic acid (DNA) sequencing. Every organism demonstrates different DNA sequences with specific nucleotides. It reveals the genetic information carried by a particular DNA segment. Nucleic acid sequencing expresses the evolutionary changes among organisms and revolutionizes disease diagnosis in animals. This paper proposes a generative adversarial networks (GAN) model to create synthetic nucleic acid sequences of the cat genome tuned to exhibit specific desired properties. We obtained the raw sequence data from Illumina next generation sequencing. Various data preprocessing steps were performed using Cutadapt and DADA2 tools. The processed data were fed to the GAN model that was designed following the architecture of Wasserstein GAN with gradient penalty (WGAN-GP). We introduced a predictor and an evaluator in our proposed GAN model to tune the synthetic sequences to acquire certain realistic properties. The predictor was built for extracting samples with a promoter sequence, and the evaluator was built for filtering samples that scored high for motif-matching. The filtered samples were then passed to the discriminator. We evaluated our model based on multiple metrics and demonstrated outputs for latent interpolation, latent complementation, and motif-matching. Evaluation results showed our proposed GAN model achieved 93.7% correlation with the original data and produced significant outcomes as compared to existing models for sequence generation.

## 1. Introduction

Deoxyribonucleic acid (DNA) sequencing is the mechanism of determining the arrangement of nucleotide bases in a fragment of DNA. Each strand in the DNA is composed of four complementary nucleotides named adenine (A), cytosine (C), guanine (G), and thymine (T) [1]. These nucleic acid sequences ascertain the scientists about detailed genetic information and help with different aspects of research. Genomic sequencing plays an essential role in understanding evolutionary relationships between different species [2]. How different life forms differ at the molecular level can be extracted from genome sequencing. It can also help track strains of infections, effects of mutation, and the root for any disease [3]. Thus, nucleic acid sequencing can potentially increase the ability to proactively identify disease before its development and commence treatment plans for the future.

Machine learning has shown promising results in clinical diagnostics. It has been proved to be well suited for solving specific tasks for clinical genomics, variant calling, phenotype-to-phenotype mapping, genome annotation, and variant classification [4]. Generative adversarial networks (GAN) since its invention has been used widely in different field of applications. Machine learning algorithms require a large amount of data for training purposes. Genomic data or any medical data have confidentiality concerns and is less in availability. Therefore, it is necessary to create synthetic data to use for research purposes. This paper proposes a generative adversarial networks (GAN) model to create nucleic acid sequences of the cat genome. This research can be helpful for disease diagnosis or clinical examination for cats and be extended for gene discovery in other species. The main contribution of this paper is as follow:

- We prepared a new dataset consisting of fecal samples collected from eight cats belonging to four categories according to their health condition. The categories are normal, dermatopathy, gastrointestinal and urology.
- The advantage of the proposed model is that it includes two separate networks in the GAN architecture itself. We propose a GAN model consisting of a predictor and an evaluator to generate synthetic nucleic acid sequences tuned to acquire desired properties of a DNA sequence. The inclusion of the predictor and the evaluator contributes to the generation of realistic samples more efficiently than existing GAN models.
- We built a predictor that is trained to classify and recognize promoter sequences in the generated synthetic sequences. This helps to tune the sequences to have properties of the promoter.
- We also implemented an evaluator that matches motif sequences in the generated synthetic data. This model helps in tuning the synthetic sequences to exhibit properties of a motif.
- Our proposed model achieves a mean correlation coefficient of 93.7%, 2% more than our previously proposed TGAN-skip-WGAN-GP model and 6–10% more than CWGAN-GP, TGAN-WGAN-GP, and TGAN-skip models.

Generative Adversarial Networks (GANs) are becoming increasingly popular in producing realistic new samples of data since first introduced [5]. The min-max function of generator and discriminator plays a significant role in achieving this. Another variation of GAN known as Conditional GAN (cGAN) was proposed later that generates new samples based on a condition that labels are discrete [6,7], images [8,9] and texts [10,11]. Until then, many variations of cGAN are proposed across different domains to improve the performance of the conditional generation of samples such as StarGAN. StarGAN [12] aims to introduce domain classification along with the single validity in the discriminator. Wasserstein GAN (WGAN) introduces a substitute method for the generator’s training that brings an improved approximation of the data distribution in the training data [13]. WGAN-GP [14] also does the same and aims to achieve the same purpose as of WGANs. Here, Wasserstein distance is used instead of KL-divergence [15]. To enhance the generative performance of GAN, Variational Autoender (VAE) was introduced [16].

GAN has been used in pretty much every domain of study and hence used in sequence generation too. GAN has also been introduced to sequence generation. In [17], a unique GAN model, referred as StoryGAN is proposed that relies on sequential cGAN framework and it generates image sequences from story paragraphs. StoryGAN uses deep Context Encoder to vigorously record the flow of the story, two discriminators where one is used at the story level and the other one is used at the image level. These two discriminators help to improve the quality of the images and reliability of the sequences produced. Kwok Tai Chui et al. proposed an improved cGAN based deep support vector machine to predict the performance of students when supported by school of family tutoring [18]. The proposed work achieved 0.968 specificity, 0.971 sensitivity and 0.954 area under the receiver operating characteristic curve (AUC-ROC). In another study [19], an integrated GAN model is proposed to forecast future frames. The primary concept is to train once generator for bi-directional prediction model to forecast previous and upcoming frames. Here, two discriminators are used that discover fake frames and also differentiated a real image sequence from a fake image sequences. In a study [20], the WGAN [21] and WGAN-GP [14] are integrated with Recurrent Neural Networks (RNNs) to make a model that learns and produce realistic sentence sequences. There are some other models that are proposed to solve the differentiation issue is the generator such as SeqGAN [22]. SeqGAN uses a stochastic policy in reinforcement learning to model the generator. StepGAN [23] is an enhancement to SeqGAN that evaluates the completely generated sequences however, it also adds to StepGAN by evaluating sub-sequences as well. Besides text, and images GANs are also used for sequence generation in genes and DNAs. A novel model is proposed in [24], referred as feedback GAN (FBGAN). As name suggests, it uses a feedback-loop framework that uses an external analyzer to enhance the synthetic gene sequences. Burak Yelmen et al. proposed a combination of GANs and restricted Boltzmann machines (RBMs) to learn the complex distribution of human genomes [25]. MichiGAN explores single-cell gene expression data and combines the advantages of variational autoencoders and GANs to generate data from disentangled representations [26]. The proposed work learns from 3 large single-cell RNA sequence datasets and uses MichiGAN to generate data from the representations.

The rest of the paper is divided as follows: Section 2 presents the proposed methodology, which is further divided into subsections describing the dataset, data preprocessing, and generative adversarial networks; Section 3 provides the results of the proposed GAN model, including the performance of predictor and evaluator; Section 4 concludes the manuscript.

## 2. Proposed Methodology

In this section, we first describe the dataset we have used for our experiment, then proceed with the overall architecture explanation and the implementation details of each model. We give an overview of the generator and discriminator architecture of the GAN model and define how we built the predictor and evaluator for our proposed architecture to enhance the quality of the generated synthetic data.

### 2.1. Dataset

Our dataset consists of fecal samples collected from eight cats belonging to four categories according to their health condition. Therefore, for every category, we collected samples from two cats. Cats who doesn’t have any health problems are named as norm_1 and norm_2. Cats with derma or skin issues have been named derm_3 and derm_4. Cats facing gastrointestinal problems are categorized as gast_5 and gast_6 and cats with urologic disease has been defined as urol_7 and urol_8. The raw DNA sequences of these cats have been prepared through Illumina next-generation sequencing (NGS) [27] which comprises of four parts, i.e., sample preparation, library construction, sequencing and preparation of raw data. NGS is an advanced sequencing technology with high scalability and speed in determining the order of the nucleotides in the whole genome or specific regions of DNA or RNA. NGS has made it possible for the biological science labs to perform and study biological systems and various related applications in-depth and efficiently. Traditional DNA sequencing does not provide the depth of information required by complex genomic studies. NGS has become a readily available tool that fills the gap through:

- Innovative sample preparation
- Rapid sequencing of whole genomes
- Targeted sequencing
- RNA sequencing that eliminates the expense and inefficiency of traditional technologies
- Studying tumor subclones and somatic variants through cancer sample sequencing
- Epigenetic factor analysis such as DNA-protein interactions
- Human microbiome study and novel pathogen identification.

The initial step is sample preparation, where DNA/RNA was extracted from the eight cats and was processed for quality control (QC). The qualified samples were passed for library construction. The next step is the library construction, where the “Herculase II Fusion DNA Polymerase Nextera XT Index Kit V2” library kit was used for our experiment, and the library protocol used was “16S Metagenomic Sequencing Library Preparation Part #15044223 Rev. B”. The sequencing library was prepared through random fragmentation of the DNA or cDNA sample, followed by 5${}^{\prime }$ and 3${}^{\prime }$ adapter ligation. Alternatively, “tagmentation” combined the fragmentation and ligation reactions into a single step that significantly increased the efficiency of the library preparation process. Adapter-ligated fragments were then PCR amplified and gel purified. For sequencing, the library was loaded into a flow cell for cluster generation. In a flow cell, fragments are captured on a lawn of surface-bound oligos complementary to the library adapters. Each fragment was amplified into distinct, clonal clusters through bridge amplification. When cluster generation was complete, the templates were ready for sequencing. Sequencing was done through Illumina SBS technology. The base calls binary is converted into FASTQ utilizing illumina package bcl2fastq. These FASTQ files contain the raw data or sequences that have been used for training GAN models to generate synthetic nucleic acid sequences of cat genome.

These raw data produce a large number of reads or sequences that contain essential and relevant genomic data. But, the sequences consist of artifacts, and therefore preprocessing is a mandatory step for further analysis of the data [28]. GC, AT, Q20 and Q30 has been obtained from the Illumina Sequencer that produces the base reads through an integrated primary real time analysis (RTA) software. Table 1 presents the statistics of the raw data containing:

- Sample ID: Sample name.
- Total read bases: Total number of bases sequenced.
- Total reads: Total number of reads. For Illumina paired-end sequencing, this value refers to the sum of read 1 and read 2 (Paired-end).
- GC(%): GC content.
- AT(%): AT content.
- Q20(%): Ratio of bases that have phred quality score of over 20.
- Q30(%): Ratio of bases that have phred quality score of over 30.

### 2.2. Proposed Generative Adversarial Networks Model for Creating Synthetic Nucleic Acid Sequences

**Figure 1:**
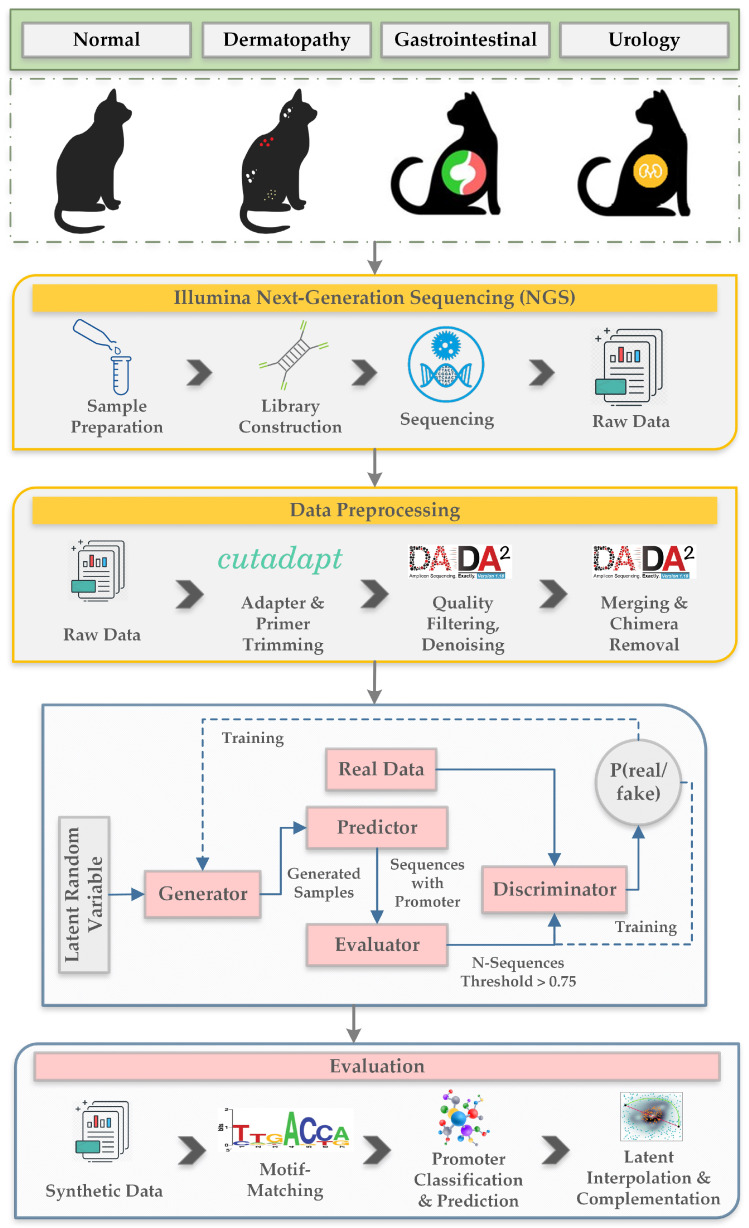
Overview of the proposed model.

The collection of raw data is followed by data preprocessing where the raw data is processed for adapter and primer trimming, denoising, quality filtering and merging and chimera removal. The processed raw data or nucleic acid sequences then proceed to the GAN model as input. The generator of the GAN model generates synthetic samples which are then passed through the predictor to predict the presences of promoter in the sequences. Sequences with promoters can only proceed to the evaluator which evaluates and scores the sequences on the basis of motif-matching. Sequences with scores above threshold are then send to the discriminator which receives both real data and tuned generated data. The discriminator then tries to identify whether the sample is from real or generated distribution. This way the GAN model creates synthetic nucleic acid sequences that are tuned to have certain desired properties. The synthetic data is then evaluated on the basis of motif-matching, latent interpolation, latent complementation and promoter classification and prediction. Figure 1 presents the overview of the proposed model.

#### 2.2.1. Data Preprocessing

In the data preprocessing section, we take the raw data as input and perform adapter trimming since adapter sequences can interfere with further analysis of the data. We also perform primer trimming to eliminate low quality data from the sequences to extract the high quality meaningful part from the sequencing data that is required for our experiment. We have used cutadapt [29] for performing adapter and primer trimming. We removed sequence errors through DADA2 [30] denoising and filtered sequences with low quality score. filterAndTrim(…) function was used to filter paired reads. In the function, we removed the first few nucleotides using trimLeft. We truncated paired reads using truncLen and filtered out all paired reads with more than maxN = 0 ambiguous nucleotides. We have merged all the paired reads using mergePairs(…) function and eliminated those pairs of reads that couldn’t be matched. Chimeras are basically a combination of reads from two samples generating or resulting a spurious amplicon. Therefore, it is necessary to remove incomplete amplified sequences that primes another sequence to generate spurious sequences. We do this through removeBimeraDenovo(…) function in DADA2. Table 2 shows the trimmed and filtered read counts for sample from each category.

#### 2.2.2. Generative Adversarial Networks

Ian Goodfellow et al. [5] in the year 2014 first introduced a machine learning framework called generative adversarial networks (GAN). The GAN architecture comprises of two neural networks named as generator (*G*) and discriminator (*D*) which competes against each other in a zero-sum game. Since, it’s invention it has been applied in various fields and have yield incredible outcomes. The generator in the GAN architecture receives latent random variable $p_{z}\left(z\right)$ from which it tries to capture and retain the data distribution $p_{d}$ over data *x*.

The discriminator then receives samples from real data as well as samples from the generator. The task of the discriminator is to correctly evaluate whether the data is from the real or the generated distribution. The generator tries to fool the discriminator by providing realistic samples, while the discriminator tries to improve the performance of the generator until the time the samples generated by the generator cannot be distinguished from the real data. Therefore, the minmax game function can be defined as in Equation (1), where the generator tries to minimize log(1−D(G(z))).

(1) $$\min_{G}\;\max_{D}\;F\left(D,G\right)=E_{x∼p_{d}\left(x\right)}\left[logD\left(x\right)\right]+E_{z∼p_{z}\left(z\right)}\left[log\left(1-D\left(G\left(z\right)\right)\right)\right]$$

In this paper, we propose a GAN model to generate synthetic nucleic acid sequences of cat genome and include a predictor and an evaluator to tune the sequences to have specific desired properties. The predictor and evaluator has been included to create synthetic sequences that are more realistic and possess properties that are similar to original cat genome sequences. After thorough experimentation, we have used Wasserstein GAN with gradient penalty (WGAN-GP) in our proposed model [13]. Training of GAN model with the loss function of Vanilla GAN is often complicated, with poor convergence rate. It suffers from model collapse problem and makes it difficult to judge whether the generator has converged, is trying to converge or it has stopped the learning process. WGAN unlike Vanilla GAN minimizes the Earth Mover also known as Wasserstein distance, between distribution of real and generated data. The goal of WGAN is to optimize the Wasserstein-1 distance and to develop a powerful discriminator that generates a relevant gradient for the generator overlooking the generator’s poor performance. Wasserstein-1 distance can be defined with minute alteration in the GAN objective as shown in Equation (2).

(2) $$\min_{G}\;\max_{D}\;E_{x∼p_{d}}\left[D\left(x\right)\right]-E_{z∼p_{z}}\left[D\left(G\left(z\right)\right)\right]$$

where *D* is the k-Lipschitz function. In WGAN, an optimal discriminator is defined by the critic and WGAN requires weight clipping of the critic in a compact space [−c,c]. This weight clipping leads to optimization problems and vanishing or exploding gradient problems. To overcome these issues, Gulrajani et al. [14] introduced WGAN-GP where they formulate a different approach to impose the Lipschitz con-straint. WGAN-GP replaces the critic weight clipping mechanism of WGAN and complies with the condition of 1-Lipschitz. Gulrajani et al. proposed that if the gradient norm of a differentiable function is one almost everywhere, it can be considered as 1-Lipschitz. Objective for WGAN-GP can be defined as the combination of the original critic loss and the gradient penalty loss as mentioned in Equations (3) and (4)

(3) $$CriticLoss_{Orig}=E_{\tilde{x}∼p_{d}}\left[D\left(\tilde{x}\right)\right]-E_{x∼p_{r}}\left[D\left(G\left(x\right)\right)\right]$$

(4) $$Penalty_{Gradient}=\lambda E_{\hat{x}∼p_{\hat{x}}}[(∥\nabla_{\hat{x}}D\left(\hat{x}\right){∥}_{2}-1{)}^{2}]$$

where $\hat{x}∼p_{\hat{x}}$ represents random samples, $p_{d}$ and $p_{r}$ defines data, and distribution of generator and λ, has been selected as 12 according to our experiments.

**Figure 2:**
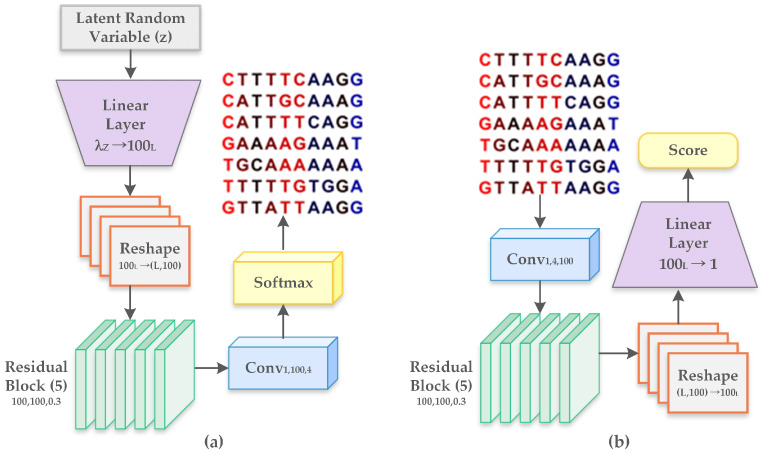
(**a**) Architecture of the Generator and (**b**) Architecture of the Discriminator.

The generator and the discriminator follows the architecture of WGAN-GP as shown in Figure 2. The generator and the discriminator uses a residual architecture. In Figure 2 *L*, denotes the length of nucleic acid sequence. The latent space dimension for generator has been defined as $\lambda_{Z}=100$. We perform sampling from the latent space based on the standard normal distribution and for every update of the generator, we execute five discriminator updates. The abbreviations mentioned in Figure 2 for designing the model layers are described below [31]:

- Linear layer $\left(\lambda_{in}\to \lambda_{out}\right)$: It represents the multiplication by a weight matrix of shape $\left(\lambda_{in},\lambda_{out}\right)$ and includes a bias parameter of dimension $\lambda_{out}$.
- Reshape $\left(Res_{1}\to Res_{2}\right)$: Altering shape of features from $Res_{1}$ to $Res_{2}$.
- Residual block $\left(L,C_{in},C_{out},r\right)$: It consists of 2 internal layers, with every layer comprising of a rectified linear unit (ReLU). It is followed by a transformation of one-dimensional convolution operation $Conv_{L}$ with filters of length *L* that is mapped from $(C_{in}$ to $C_{out}$ channels. The output from the second layer is multiplied by r≤1 and forwarded to the input of the residual block.
- Convolutional layer $\left(L,C_{in},C_{out}\right)$: Refers to one-dimensional convolution with filters of length *L*, mapped from $C_{in}$ to $C_{out}$ channels and followed by an inclusion of bias parameter of dimension $C_{out}$.
- Softmax: Softmax is the final activation function representing four nucleotides with dimension four. It is applied to each position separately along the sequence length.

Promoters are defined as subsequences or small DNA sequences that is located in the beginning of the gene transcription site and initiates transcription of specific genes in the genome. It is required to turn the gene on or off and thus considered as one of the most important factor for sequence generation. The proposed predictive model is evaluated on the promoter sequence of *E. coli* [32,33]. To evaluate our proposed model, we have used three sub-types for promoter sequence i.e., sigma−24, sigma−32 and sigma−54. DNA sequences consists of four nucleotides (A, T, C and G). The sequences are first converted to numeric form through one-hot encoding. The four element vector for each nucleotide is represented as:

$$\begin{array}{c} A(1,0,0,0)\\ T(0,1,0,0)\\ C(0,0,1,0)\\ G(0,0,0,1) \end{array}$$

**Figure 3:**
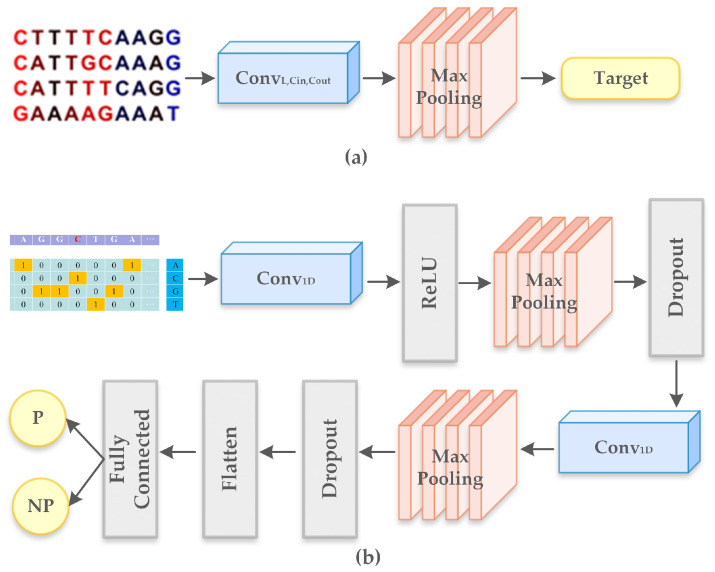
(**a**) Proposed model for evaluator and (**b**) Proposed model for predictor.

Our proposed layered-CNN model predicts the presence of a promoter in the generated sequences and identifies three subtypes of the promoter. The predictor has been built to tune the generated sequences to have the desired properties of a promoter.Only sequences predicted with promoter is forwarded to the evaluator. Figure 3b shows the architecture of the proposed predictor model. Our proposed model contains two one-dimensional convolutional layers. Both the convolutional layers are followed by a ReLU activation function, max pooling with a window size of 6 and a dropout layer with 0.5 probability. The features are flattened through flatten layer. The fully connected layer then classifies the promoters. Sequences with the presence of promoter is forwarded to the evaluator.

Figure 3 represents the architecture of the proposed model for predictor and evaluator. We built an explicit evaluator which encodes a known motif and evaluates whether the sequences from the predictor exhibit the motif properties and comprises the sequences of the motif. Motifs are short recurring subsequences patterns in DNA that are assumed to obtain biological functions and indicate binding sites for proteins. Motif of length Len can be represented by a Len×4 position weight matrix (PWM) where each column represents the probability distribution of the frequency of each nucleotide. Figure 3a describes the architecture of the evaluator. We use an one-dimensional convolution which computes the inner product of PWM with each Len subsequence of the generated data. We then perform max-pooling to select a single convolutional output that has the highest value. This helps us generate the final score for the sequences obtained from the predictor.

Sequences generating atleast a single subsequence will have a high score. The threshold is set to 0.75. We used a grid-search approach with a selected range from 0.65 to 0.9 with a step size of 0.05. We experimented with 0.65, 0.70, 0.75, 0.8 and 0.9 threshold values. Keeping the threshold value to 0.75 could generate higher number and more accurate realistic sequences than other threshold values. Sequences with scores greater than the threshold are passed to the discriminator. The discriminator then tries to distinguish and identify correctly between real and synthetic sequences. The training continues until the generator and the discriminator reaches Nash equilibrium.

## 3. Results

In this section, we present the evaluation results of our proposed model. We evaluated various existing GAN models and provided the performance comparison of six GAN models on generating the synthetic nucleic acid sequences. The models we have used for comparison are TGAN [34], CTGAN [35], TGAN-skip [36], TGAN-WGAN-GP [37], CWGAN-GP [38] and TGAN-skip-WGAN-GP [39]. The performance comparison has been based on the quality of the synthetic data measured through mean correlation coefficient, root mean square error (RMSE), Fréchet Distance (FD), mirror column association, mean absolute error (MAE) and percent root mean square difference (PRD).

For evaluating the prediction model, we have compared the performance of our model with seven other existing models based on accuracy (Acc), Matthews correlation coefficient (MCC), sensitivity (Sen) and specificity (Spc).The models used for prediction comparison are Bidirectional long short term memory (BiLSTM), Bidirectional recurrent neural network (BiRNN), support vector machine (SVM), Ensemble, Naive Bayes, extreme gradient boosting, gradient boosting. We refer our model as the layered-CNN model.

We computed correlation between the original sequences and generated synthetic nucleic acid sequences. We have measured Pearson’s correlation coefficient which ranges from −1 to 1. The correlation values are represented in Table 3, where direct relationship is represented through positive value, inverse relationship with negative value and zero represents no relationship. We define Pearson’s correlation coefficient $\left(P_{Coef}\right)$ as in (5) [40].

(5) $$\begin{array}{c} P_{Coef}=\frac{\sum_{i=1}^{n}\left(Or_{i}-Or\right)\left(Sn_{i}-Sn\right)}{\sqrt{\left[\sum_{i=1}^{n}\left(Or_{i}-\bar{O}r\right)\right]\left[\sum_{i=1}^{n}\left(Sn_{i}-\bar{S}n\right)\right]}} \end{array}$$

where Or and Sn represents original and synthetic nucleic acid sequences.

We calculated MAE to measure the absolute error between the original and the synthetic sequences. Equation (6) defines the formulation for computing MAE.

(6) $$\begin{array}{c} MAE=\frac{1}{n}\sum_{i=1}^{n}\left|Or_{i}-Sn_{i}\right| \end{array}$$

Distortion between the original and synthetic sequences have been defined by PRD as shown in Equation (7).

(7) $$\begin{array}{c} PRD=\sqrt{100\frac{\sum_{i=1}^{n}{\left(Or_{i}-Sn_{i}\right)}^{2}}{\sum_{i=1}^{n}{\left(Or_{i}\right)}^{2}}} \end{array}$$

RMSE is computed to measure the stability of the generated sequences and to quantify how different the synthetic data is from the original data as shown in Equation (8).

(8) $$\begin{array}{c} RMSE=\sqrt{n\sum_{i=1}^{n}{\left(Or_{i}-Sn_{i}\right)}^{2}} \end{array}$$

Mirror Column association finds the association between each column of the original and the synthetic dataset. Higher value represents that the generated data has higher association indicating better performance of the model.

Fréchet Distance is computed to evaluate how similar the ordering and location of points are along the curves. Suppose $Or_{O}=a_{1},a_{2},a_{3},\ldots ,a_{O}$ is the order of points in the original curves and $Sn_{P}=d_{1},d_{2},d_{3},\ldots ,d_{P}$ is the order of points along the curve for the synthetic data, then length ∥l∥ of the sequence is measured as in (9):

(9) $$\begin{array}{c} ∥l∥=\max_{i=1,\ldots ,n}l\left(a_{s_{i}},d_{t_{i}}\right) \end{array}$$

where *l* is the euclidean distance and $a_{s_{i}}$ and $d_{t_{i}}$ are the order of points sequence. So we compute the Fréchet Distance as shown in (10) [41]:

(10) $$\begin{array}{c} FD(O,P)=\min∥l∥ \end{array}$$

We present the performance comparison of different models in Table 4 which depicts that our proposed GAN model performs the best in generating synthetic nucleic acid sequences of cat genome.

We evaluate the performance of the predictor through four different metrics namely accuracy, sensitivity, Matthews correlation coefficient and specificity. We can define these metrics through below Equations (11)–(14) [31].

(11) $$MCC=\left(TP\times TN-FP\times FN\right)/\left(\sqrt{\left(TP+FP)(TP+FN)(TN+FP)(TN+FN\right)}\right)$$

(12) $$\begin{array}{c} Acc=\frac{TP+TN}{TP+TN+FP+FN} \end{array}$$

(13) $$\begin{array}{c} Sen=\frac{TP}{TP+FN} \end{array}$$

(14) $$\begin{array}{c} Spc=\frac{TN}{TN+FP} \end{array}$$

where *TP*, *TN*, *FP* and *FN* represents true positives, true negatives, false positives and false negatives. Table 5 shows the performance comparison of different prediction models for three sigma promoters. We have adopted 10-fold cross-validation for evaluating specificity, sensitivity, AUC-ROC curve performance.

**Figure 4:**
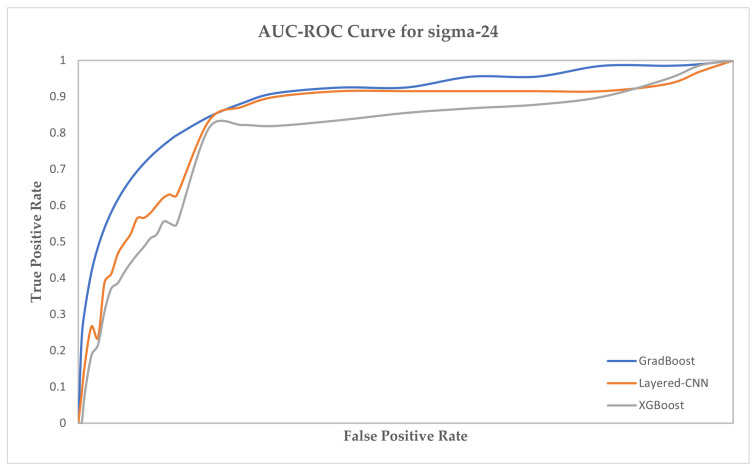
AUC-ROC Curve for sigma-24 prediction.

**Figure 5:**
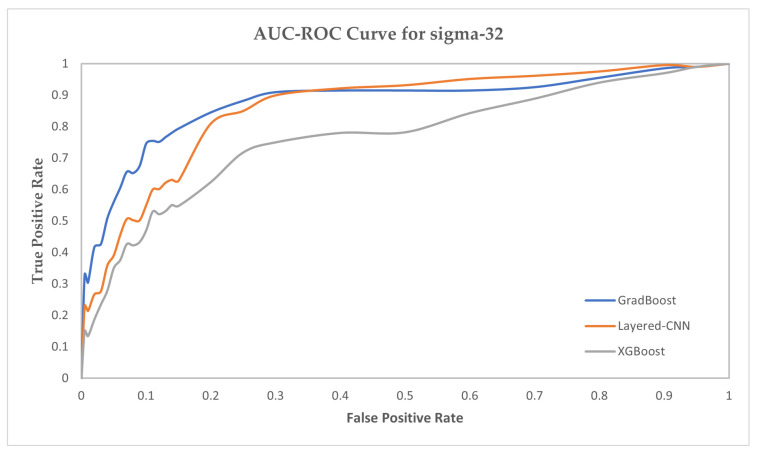
AUC-ROC Curve for sigma-32 prediction.

**Figure 6:**
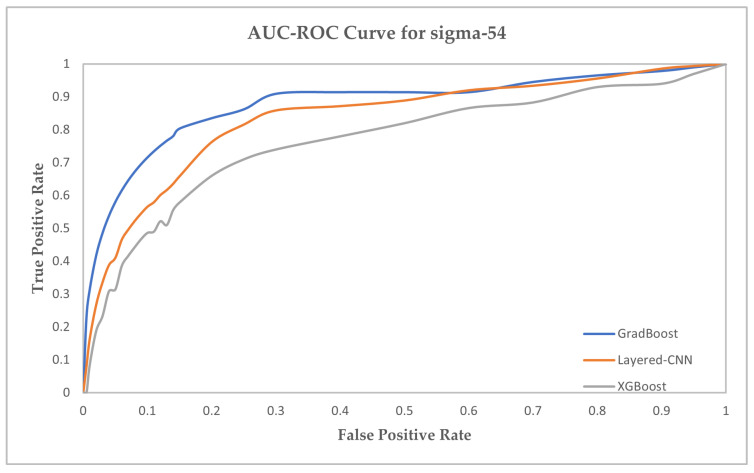
AUC-ROC Curve for sigma-54 prediction.

We have provided the AUC-ROC curve in Figure 4, Figure 5 and Figure 6 for three prediction models which performed the best in predicting the sigma promoters. The prediction models are GradBoost, Layered-CNN and XGBoost.

**Figure 7:**
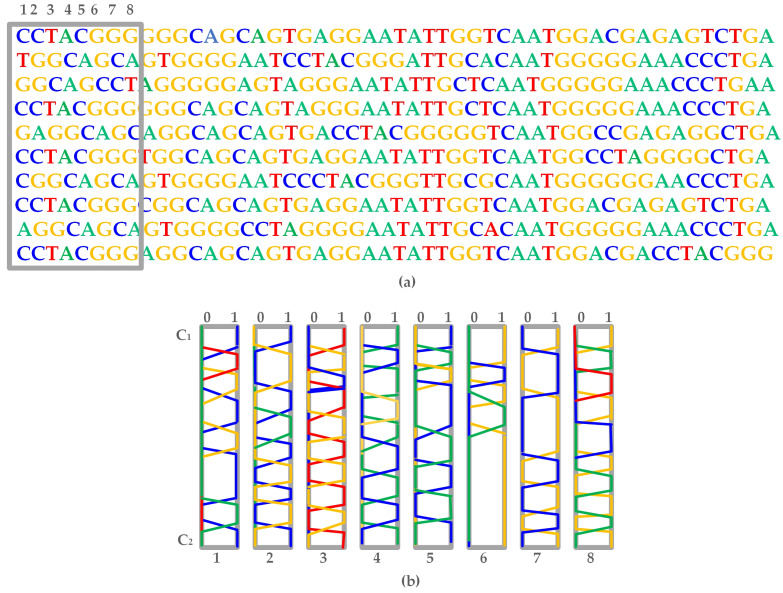
Linear interpolation between two randomly selected points $C_{1}$ and $C_{2}$. (**a**) Generated sequences, (**b**) Linear interpolation.

Next, we have evaluated our generative architecture by training it on large dataset and experimented what it has learned through manipulation of the latent space. We considered latent interpolation. Figure 7a, shows the generated sequences where A is represented through green channel, C through blue channel, G through yellow channel and T through red channel. We output the latent interpolation between two points $C_{1}$ and $C_{2}$ and represent it for the first eight position of the sequences in Figure 7b. We can see that the generator can output the approximation of the one-hot data efficiently. Interpolation of the latent values occur from top-to-bottom. Generated sequences are represented from left to right.

**Figure 8:**
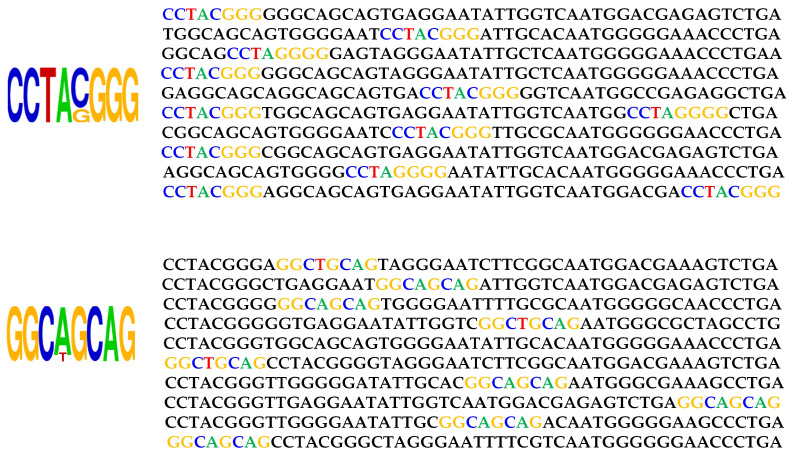
Experimentation result for motif-matching.

In Figure 8, we perform experiment for motif-matching. On the left side of the figure is the sequence logo for the PWM which was detected by our proposed evaluator. The heights of the letter represents the relative frequency at each position. Sequences possessing the motif sequences score high and is passed to the predictor. On right side of the Figure 8, we present the sample sequences that achieved high scores and indicated strong matching for the motif.

**Figure 9:**
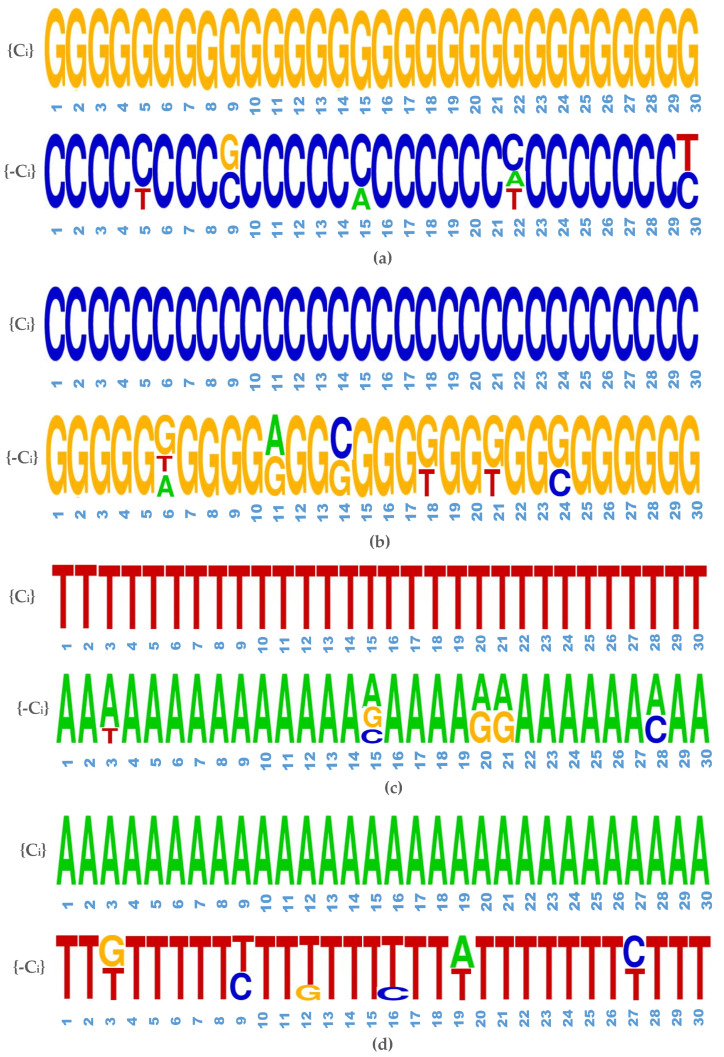
Experimentation result for latent space-complementation. (**a**) Complementary nucleotide for G, (**b**) Complementary nucleotide for C, (**c**) Complementary nucleotide for T, (**d**) Complementary nucleotide for A.

We also explored reflection in the latent space, also known as latent complementation. The complementary nucleotide are A⟺T and G⟺C. For the generation of same data through multiple latent vectors, we fix a sequence and via gradient based search find different latent points. We reflect all the latent points and the generated synthetic sequences are then decoded. From Figure 9, we can see that the reflected coordinates have strong bias for generating complementary nucleotide for each nucleotide A, T, G and C. This proves that our proposed GAN model learned nucleotide complementation efficiently and produced realistic nucleic acid sequences.

## 4. Conclusions

In this paper, we proposed a generative adversarial networks based model to create synthetic nucleic acid sequences of cat genome that are realistic and are tuned to exhibit certain desired properties. We obtain the raw sequence data through Illumina next-generation sequencing, which is then forwarded for data preprocessing. We perform adapter and primer trimming, quality filtering, denoising and merging and chimera removal. The preprocessed raw nucleic acid sequences are then passed to the GAN model, for which we use the architecture of WGAN-GP. The generator generates synthetic data which goes through the predictor to extract samples consisting of promoter sequences. The extracted sequences is then used as input to the evaluator which scores the synthetic sequences on motif-matching. Strongly matched sequences with scores higher than threshold are then filtered and sent to the discriminator for evaluation. The training continues until the generator generates sequences that cannot be distinguished from the real sequences. Our proposed GAN model can efficiently generate nucleic acid sequences of cat genome that reveals desired properties like promoter and motif.

We evaluated the quality of our generated synthetic data through motif-matching, promoter classification and prediction and latent interpolation and complementation. We have used multiple metrics such as mean correlation coefficient, RMSE, FD, MAE, mirror column association and PRD for evaluating the performance of various GAN models to generate synthetic sequences. For classifier and predictor evaluation we computed MCC, accuracy, sensitivity and specificity. We have presented results for latent interpolation, latent complementation and motif-matching. Results for all experiment shows that our proposed architecture could create synthetic nucleic acid sequences efficiently and exhibits desired properties of cat genome. We feel the proposed methodology can be expanded and implemented for genome data of other mammals. The proposed work can be extended by building a conditional GAN architecture to condition properties that would be mandatory for creating realistic sequences. Our future work would deal with the implementation of a conditional GAN architecture to enhance the generation of synthetic data and to build a single model for creating genome data for multiple species. One of the future goal is to increase the prediction accuracy and improve the model architecture for enhanced performance. Also, we would work on domain adaptation to genomic sequences through advanced generative adversarial networks models.

## Figures and Tables

**Table 1:** Statistics of Raw Data.

Sample ID	Total Read Bases (bp)	Total Reads	GC (%)	AT (%)	Q20 (%)	Q30 (%)
drem_3	54,370,232	180,632	56.84	43.16	88.21	77.60
drem_4	62,781,376	208,576	55.57	44.43	89.66	79.97
gast_5	55,694,632	185,032	53.89	46.11	90.73	81.30
gast_6	58,980,950	195,950	54.07	45.93	90.08	80.37
norm_1	68,054,294	226,094	51.64	48.36	91.34	82.10
norm_2	65,382,618	217,218	55.47	44.53	89.31	79.59
urol_7	56,866,124	188,924	53.52	46.48	91.10	81.93
urol_8	55,780,116	185,316	51.24	48.76	91.49	82.21

**Table 2:** Trimmed and Filtered Read Counts for Each Sample.

Sample Name	Adapter & Primer Trimming	Quality Filter	DenoisedFor	DenoisedRev	MergedPair	Non-Chimeric
norm_1	112,078	91,286	90,092	90,575	86,784	66,605
norm_2	107,715	84,245	82,726	83,449	78,310	59,086
derm_3	89,403	68,194	67,967	68,075	67,408	63,361
derm_4	103,322	81,146	79,791	80,394	74,674	56,874
gast_5	91,749	74,340	72,938	73,598	70,158	62,708
gast_6	97,252	76,664	75,368	75,863	71,881	52,800
urol_7	93,669	76,908	76,833	76,855	76,702	75,019
urol_8	91,949	75,438	75,150	75,197	74,187	66,993

**Table 3:** Representation of Correlation Values.

Correlation Values	Relation
0 to 0.3 or 0 to −0.3	Negligibly correlated
0.3 to 0.5 or −0.3 to −0.5	Low correlation
0.5 to 0.7 or −0.5 to −0.7	Moderately correlated
0.7 to 0.9 or −0.7 to −0.9	Highly correlated
0.9 to 1 or −0.9 to 1	Extensively correlated

**Table 4:** Performance comparison of various GAN models to generate synthetic sequences.

Model	Mean Correlation Coefficient	RMSE	FD	MAE	Mirror Column Association	PRD
TGAN	0.628	0.72	0.78	0.71	0.691	78.6
CTGAN	0.790	0.72	0.76	0.66	0.716	77.9
TGAN-skip	0.833	0.69	0.72	0.63	0.772	73.5
TGAN-WGAN-GP	0.863	0.66	0.68	0.58	0.783	71.0
CWGAN-GP	0.875	0.66	0.68	0.55	0.849	67.5
TGAN-skip-WGAN-GP	0.913	0.62	0.61	0.49	0.914	58.9
Proposed Model	0.937	0.57	0.58	0.44	0.939	53.1

**Table 5:** Performance of the predictor (layered-CNN) in the proposed GAN model compared to other existing models.

	sigma-24				sigma-32				sigma-54			
**Model**	**Acc**	**MCC**	**Sen**	**Spc**	**Acc**	**MCC**	**Sen**	**Spc**	**Acc**	**MCC**	**Sen**	**Spc**
BiLSTM	65.23	72.13	76.52	74.26	66.11	70.41	78.47	74.89	65.41	73.62	72.47	71.84
BiRNN	66.78	74.56	76.71	74.55	65.72	72.55	78.61	73.74	65.79	74.55	77.82	73.58
SVM	69.91	80.23	79.16	75.83	67.83	78.91	80.16	78.11	67.46	78.02	77.91	74.60
Ensemble	73.12	83.81	81.88	78.92	72.90	80.83	82.88	78.50	69.92	85.39	81.47	77.92
Naive Bayes	76.51	85.66	83.45	81.65	77.36	84.57	84.66	83.22	71.47	88.40	86.92	82.49
XGBoost	79.34	88.94	86.83	85.02	80.62	89.84	88.12	85.64	75.55	89.15	86.61	85.62
GradBoost	84.81	92.32	92.55	88.38	87.15	93.47	91.48	90.46	81.36	93.08	91.44	86.71
Layered-CNN	91.63	94.56	92.53	92.36	92.57	93.61	92.16	93.73	93.38	94.96	92.02	93.42
